# Formation permeability estimation using mud loss data by deep learning

**DOI:** 10.1038/s41598-025-94617-7

**Published:** 2025-04-30

**Authors:** Yaser Abdollahfard, Seyed Morteza Mirabbasi, Mohammad Ahmadi, Abdolhossein Hemmati-Sarapardeh, Sefatallah Ashoorian

**Affiliations:** 1 Petroleum Engineering Department, Amirkabir University of Technology, Tehran, Iran; 2 Department of Petroleum Engineering, Shahid Bahonar University of Kerman, Kerman, Iran; 3 Institute of Petroleum Engineering, School of Chemical Engineering, University of Tehran, P.O. Box:11155-4563, Tehran, Iran

**Keywords:** Permeability, Mud loss, Convolutional neural networks (CNN), Deep jointly informed neural networks (DJINN), Artificial intelligence, Engineering, Fluid dynamics, Geology

## Abstract

Permeability estimation plays an essential role in the assessment of reservoirs and hydrocarbon extraction. There are various methods to evaluate the formation and estimate the formation permeability, but in some cases, the evaluation may not be done or it may not be done correctly. This study focuses on a novel method to estimate the formation’s permeability with appropriate accuracy using the mud loss data. Machine learning applications are becoming more popular nowadays and can succeed in many fields. This current research focuses on the application of mud loss data and deep learning to estimate the formation’s permeability. To implement and validate our methodology, it is considered pilot cases including reservoir and drilling parameters values (depth, formation type, formation thickness, mud density, mud viscosity, and formation permeability). It is assumed that mud loss was occurred because of deferential pressure between formation pressure and bottom-hole pressure. The mud loss rate data were generated at different sets of reservoir and drilling data values using a reservoir simulator and then evaluated by calculating the correlation coefficients to ensure their validity and to check the fit under real conditions. This can be used to estimate the formation permeability values. One-dimensional convolutional neural networks(1D-CNN), a type of convolutional neural network, is utilized to be trained with data to perform a regression problem based on the contribution of flattening, dropout, and fully connected layers to estimate permeability with high accuracy (training data R^2^ = 0.970, testing data R^2^ = 0.964). Then the new deep learning method, Deep jointly informed neural network (DJINN), with the cooperation of neural networks and decision trees, provides a more accurate model than 1D-CNN (training data R^2^ = 0.978, testing data R^2^ = 0.972). These descriptions may provide new applications for mud loss data, where data while drilling can be used to predict formation permeability and provide insights for petroleum engineers to accurately measure design.

## Introduction

Formation permeability is a critical parameter in petroleum engineering, influencing fluid flow within reservoirs and impacting hydrocarbon recovery efficiency. Traditional methods for estimating permeability, such as core sampling and well testing, can be costly and time-consuming, often yielding limited spatial coverage of reservoir properties. Moreover, the inherent heterogeneity of geological formations can lead to significant discrepancies in permeability values, complicating reservoir characterization. As a result, there is an increasing need for innovative approaches that leverage advanced data analytics and machine learning techniques to enhance the accuracy and efficiency of permeability estimation from available well log data and other indirect measurements^1^.

Understanding and evaluating formation permeability is essential for several reasons:

1. Hydrocarbon flow: Formation permeability directly affects the movement of oil and gas within the reservoir. High permeability allows for easier flow of hydrocarbons to the wellbore, which is essential for efficient extraction. Conversely, low permeability can hinder production rates and increase extraction costs^1^.
2. Reservoir management: Understanding permeability is vital for effective reservoir management and development strategies. Engineers use permeability data to predict reservoir behavior under various production scenarios, optimize well placement, and enhance recovery techniques^2^.
3. Enhanced oil recovery (EOR): In enhanced oil recovery methods, knowledge of formation permeability is crucial for selecting appropriate techniques, such as water flooding or gas injection. The effectiveness of these methods often depends on the permeability characteristics of the reservoir rock^3^.
4. Modeling and simulation: Accurate permeability measurements are essential for creating reliable reservoir models and simulations. These models help engineers forecast production performance, evaluate the economic viability of projects, and make informed decisions regarding drilling and completion strategies^4^.

Therefore, the precise measurement of this parameter is of utmost importance. The prediction of reservoir permeability has been of key interest in the industry as investment decisions based on the volume of hydrocarbon resources are dependent on their accuracy^5^. As a result, considerable time and money have been allocated to take advantage of technological advances in data collection from cores and well testing, among other activities, to help reduce the permeability data uncertainty and Improve the reservoir performance prediction^6^. On the other hand, such conventional methods often face problems such as a lack of data in some regions that makes it impossible to determine formation permeability.

Machine learning (ML) is a subset of artificial intelligence (AI) that focuses on the development of algorithms and statistical models that enable computers to perform tasks without explicit programming. Instead of being programmed with specific instructions, ML systems learn from data, identifying patterns and making decisions based on that information. In the past decade, the advancements in computer science especially in the field of artificial intelligence and machine learning (ML) enabled us to effectively extract basic data from real-world data collected in oil industry applications and employ them for better reservoir characterization^7–10^. ML is broadly acknowledged to improve our understanding of wells^11^, production, and reservoir areas^12^. In specific, ML is most broadly utilized in reservoir management and has accomplished important outcomes such as permeability, porosity, and tortuosity prediction^13^, modeling CO_2_-oil systems minimum miscibility pressure^14^, shale gas production forecast^15^, reservoir characterization^16^, predicting formation damage of oil fields^17^, digital 3D core reconstruction^18–20^, well test interpretation^21,22^, shale gas production optimization^23,24^, well log processing^25^, modeling wax deposition of crude oils^26^ and history matching^27,28^. This has motivated numerous researchers to gradually abandon the use of multiple linear regression models and empirical correlations in favor of incorporating ML when forecasting significant reservoir petro-physical properties^29^.

### ML approach for permeability prediction

Artificial neural network (ANN) is a famous approach that uses the obtained results to predict permeability^30^. In recent years, some authors have studied reservoir characterization problems from different aspects, including soft computing methods ^31–33^. The results of such studies revealed that soft computing models outperform regression models. The advantage of computational methods over regression lies in the fact that elemental uncertainties or heterogeneities are not explicitly included in computational regression methods^32^.

Permeability can be evaluated by interpreting in situ measurements taken by formation testers using well-testing equipment and well-logging. During verification, given the average permeability thickness, transient well testing provides a wealth of information about the flow capacity of the reservoir. Another worthwhile method for measuring the absolute permeability of reservoirs is to conduct flow experiments using representative core samples^34^. Geoscientists can therefore manage the production process effectively with the help of a reliable and accurate permeability estimation.

Several studies have been proposed to estimate permeability. Mohagueg et al.^35^ presented their three main approaches to permeability estimation, including analytical, statistical, and computational tools using well-log data. Chehrazi and Rezaee^36^ introduced a classification plan for permeability prediction models, including analytical models, soft computing models, and porous phase models using well-log data. Rezaee et al.^37^ presented the results of a research project that investigated permeability prediction for the Precipice Sandstone of the Surat Basin which machine learning techniques were used for permeability estimation based on multiple wireline logs. Tembely et al.^38^ emphasize the important role of feature engineering in predicting physical properties using machine and deep learning. The proposed framework, which integrates various learning, rock imaging, and modeling algorithms, is capable of rapidly and accurately estimating petrophysical properties to facilitate reservoir simulation and characterization. Okon et al.^39^ presented an ANN model to forecast the physical properties of reservoirs namely, porosity, permeability, and water saturation, developed based on logs from fifteen fields. A joint reversal technique based on a multilayer linear calculator and particle swarm optimization algorithm was applied by Yasin et al.^40^ to estimate the spatial variation of important petrophysical parameters e.g. porosity, permeability, and saturation, and essential geo-mechanical specifications (Poisson’s ratio, and Young’s modulus) for downhole zones using seismic data. Anifowose et al.^41^ conducted stringent parametric research to examine the comparative accuracy of ML techniques in estimating the permeability of the carbonate reservoir in the Middle East using integrating seismic attributes and wireline data. Akande et al.^42^ studied the predictability and impact of feature engineering on the precision of support vector machines in estimating carbonate reservoir permeability using well-log data. Bruce et al.^43^ accomplished ANN to process permeability estimation by the usage of wireline logs. El Ouahed et al.^44^ proposed combining the ANN with fuzzy logic to fully account for the fractured reservoir using well-log data. Al Khalifah et al.^45^ used ANN and genetic algorithms to estimate cores permeability measured by lab experiments.

The contents mentioned in the previous paragraph are analyzed in Table 1 which provides a comparison between the research of different researchers on permeability estimation, where the method and type of data are given. It can be seen that the researchers used statistical tools and artificial intelligence to estimate the permeability of the formation, and in their research, the data used included well log data, rock imaging data, seismic data, and core data.

**Table 1:** Tools and data types used by researchers to estimate permeability.

Item	Researcher	Tool(s)	Data type
1	Mohagueg et al.^35^	Analytical, statistical, and computational tools	Well log data
2	Chehrazi and Rezaee^36^	Analytical models, soft computing models, and porous phase models	Well log data
3	Rezaee et al.^37^	ML techniques	Multiple wireline logs data
4	Tembely et al.^38^	Machine and deep learning	Rock imaging data
5	Okon et al. ^39^	ANN	Well log data
6	Yasin et al.^40^	A joint reversal technique based on a multilayer linear calculator and particle swarm optimization algorithm	Seismic data
7	Anifowose et al.^41^	ML techniques	Integrating seismic attributes and wireline data
8	Akande et al. ^42^	Support vector machines	Well log data
9	Bruce et al. ^43^	ANN	Wireline logs data
10	El Ouahed et al. ^44^	Combining the ANN with fuzzy logic	Well log data
11	Al Khalifah et al.^45^	ANN and genetic algorithm	Core data

### Lost circulation vs. formation permeability

**Fig. 1:**
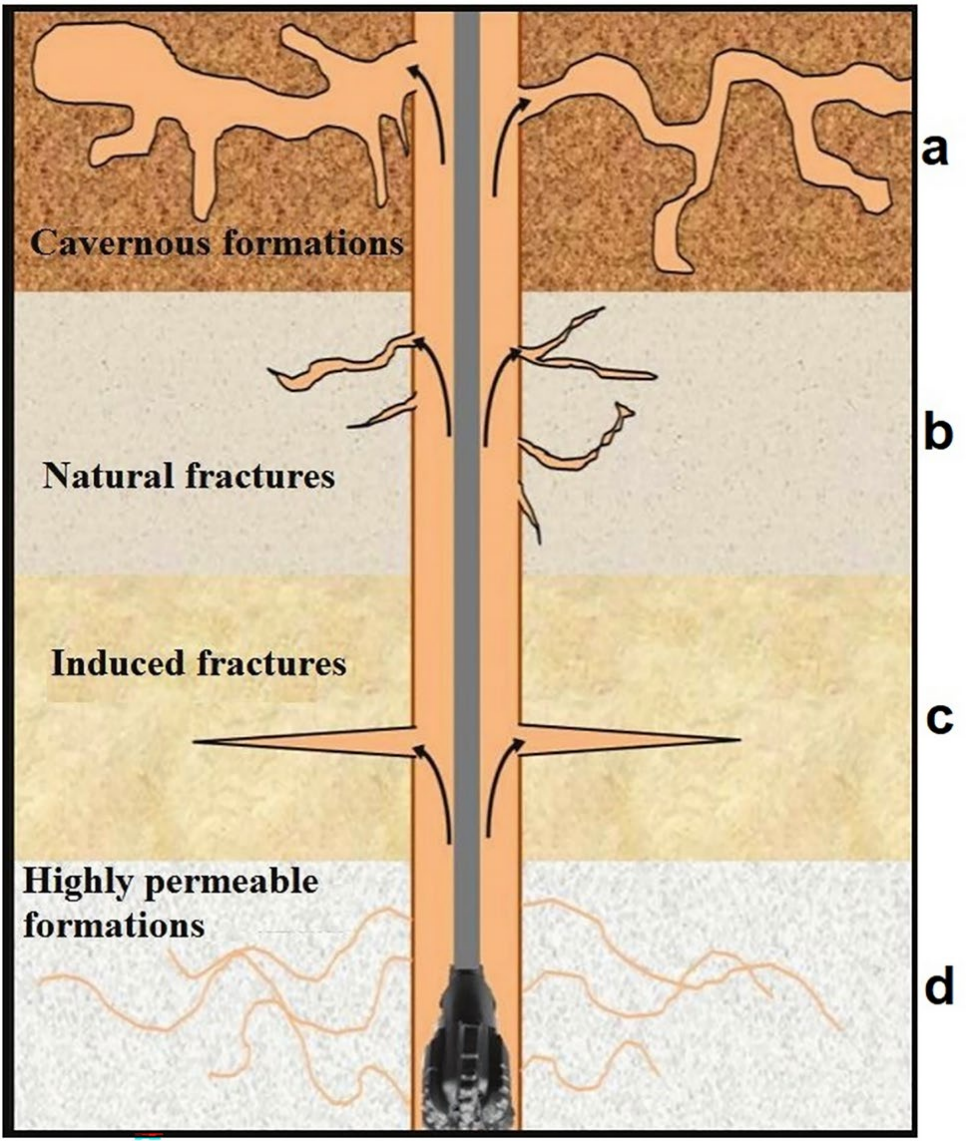
Schematic classification of lost circulation^51^.

Lost circulation is a prevalent drilling problem, especially in formations with high permeability, and natural or induced fractures ^46,47^. Lost circulation can occur in a variety of formations ranging from h shallow, unconsolidated geological layers to well-consolidated geological layers which are disrupted by drilling fluids hydrostatic pressure ^48,49^. Two conditions are necessary for a loss of circulation in the borehole to occur. First the pressure at the bottom of the well exceeds the pore pressure and next there should be a fluid flow path for lost circulation^50^. Underground routes that cause to occur lost circulation can be defined as following classes:

- **Cavernous formations:** In the direction of drilling in some formations, there are cavernous and empty spaces in which, as a result of drilling the formations, a large amount of mud loss occurs(Fig. 1a).
- **Natural fractures:** The existence of a natural fracture network, which is created by tectonics in the formation, can act as a conduit to cause leakage in the formation, and the amount of leakage depends on several factors which are mentioned below(Fig. 1b).
- **Induced fractures (e.g. quick tripping or blowouts):** In this mechanism, as a result of drilling operations such as tripping and blowouts, the bottom hole pressure increases, and cracks are created by induction. In fact, due to the low strength of some formations against stress, due to the application of additional stresses on the formation, fracture occurs in the formation(Fig. 1c).
- **Highly permeable formations:** The presence of permeable formations causes a large amount of drilling fluid to leak into the formation due to the pressure difference between the bottom hole and the formation pressure(Fig. 1d).

Fractures are an important cause of drilling fluids loss to formations whose lost circulation severity depends on fracture opening width, fracture density, fracture orientation, fracture distribution, fracture network, etc.^52–54^.

The loss ratio indicates paths of lost circulations and can show what remedial technique should be employed to counteract the loss. The lost circulation severity can be divided into four classes as follows ^55–57^:

- Seepage losses: less than 1 m^3^/h.
- Partial losses: 1–10 m^3^/h.
- Severe losses: more than 15 m^3^/h.
- Complete losses: no fluid comes out of the annulus.

In this study, for the first time, a novel method is proposed to have another important use of mud loss data. To this end, synthetic data driven from a commercial reservoir simulator is used as input to train and build our AI model. Mud loss data which is generated by a simulator is used to estimate formation permeability using Deep Jointly Informed Neural Networks(DJINN) and Convolutional Neural Networks(CNN) by formation type, formation thickness, mud density, mud viscosity, drilling depth, and mud loss rate data, which is presented as an accurate prediction of formation permeability.

## Methodology

**Fig. 2:**
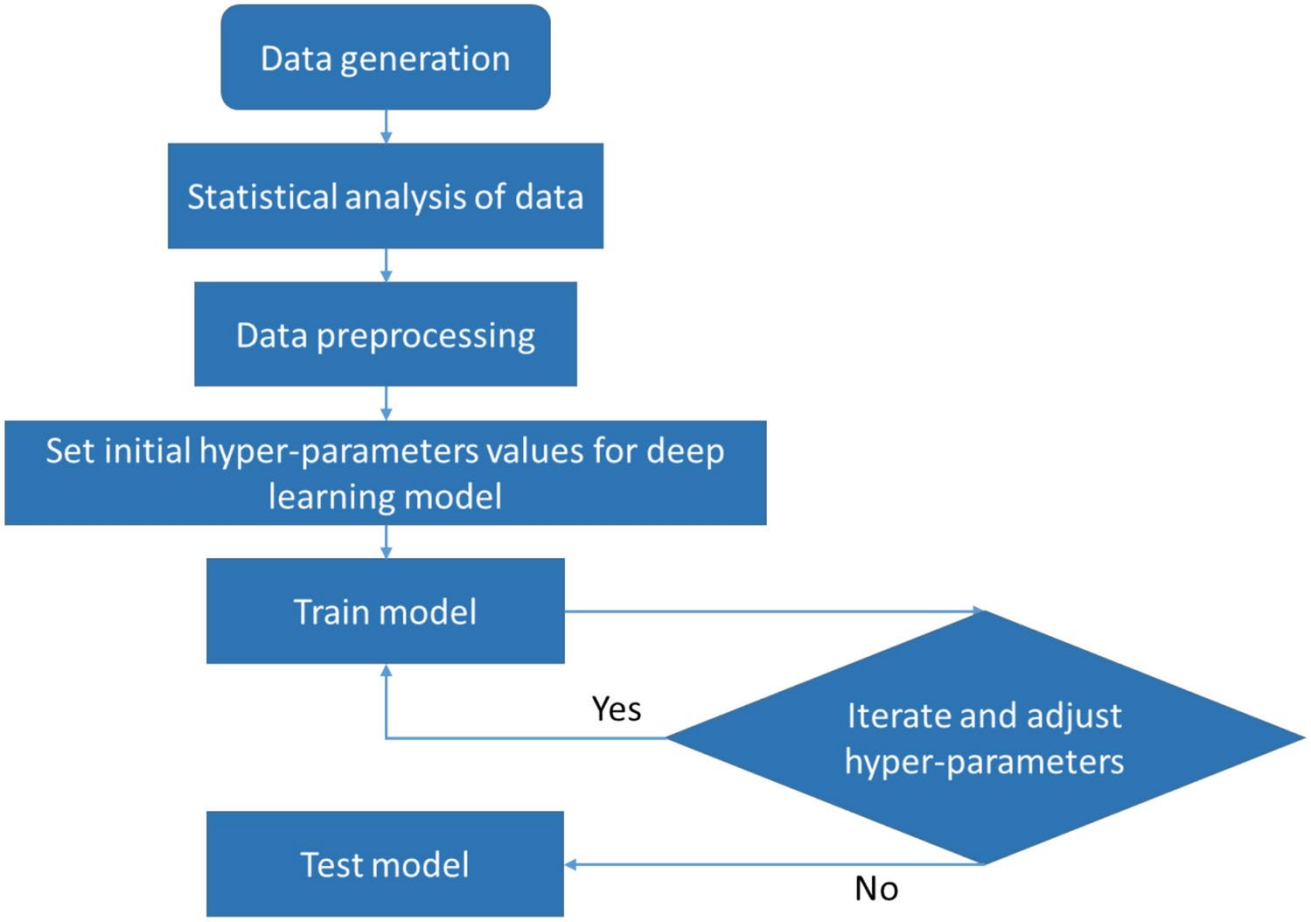
Deep learning model development flowchart.

The model development diagram is shown in Fig. 2 and the method preparation is discussed in Sects. 2.1 to 2.5. The model development flowchart begins with data generation where all of the parameters related to mud loss are generated. The generated data is then subjected to statistical analysis. Next, the data undergoes preprocessing to make it suitable for modeling. The modeling phase begins with initializing the hyper-parameters for the deep learning model. When hyper-parameters are initialized, the models are trained with an adaptive moment estimation (Adam) optimizer. The hyper-parameters are adjusted and iterated using the trial-and-error method until the model shows good performance metrics with a minimum error.

### Data generation

**Fig. 3:**
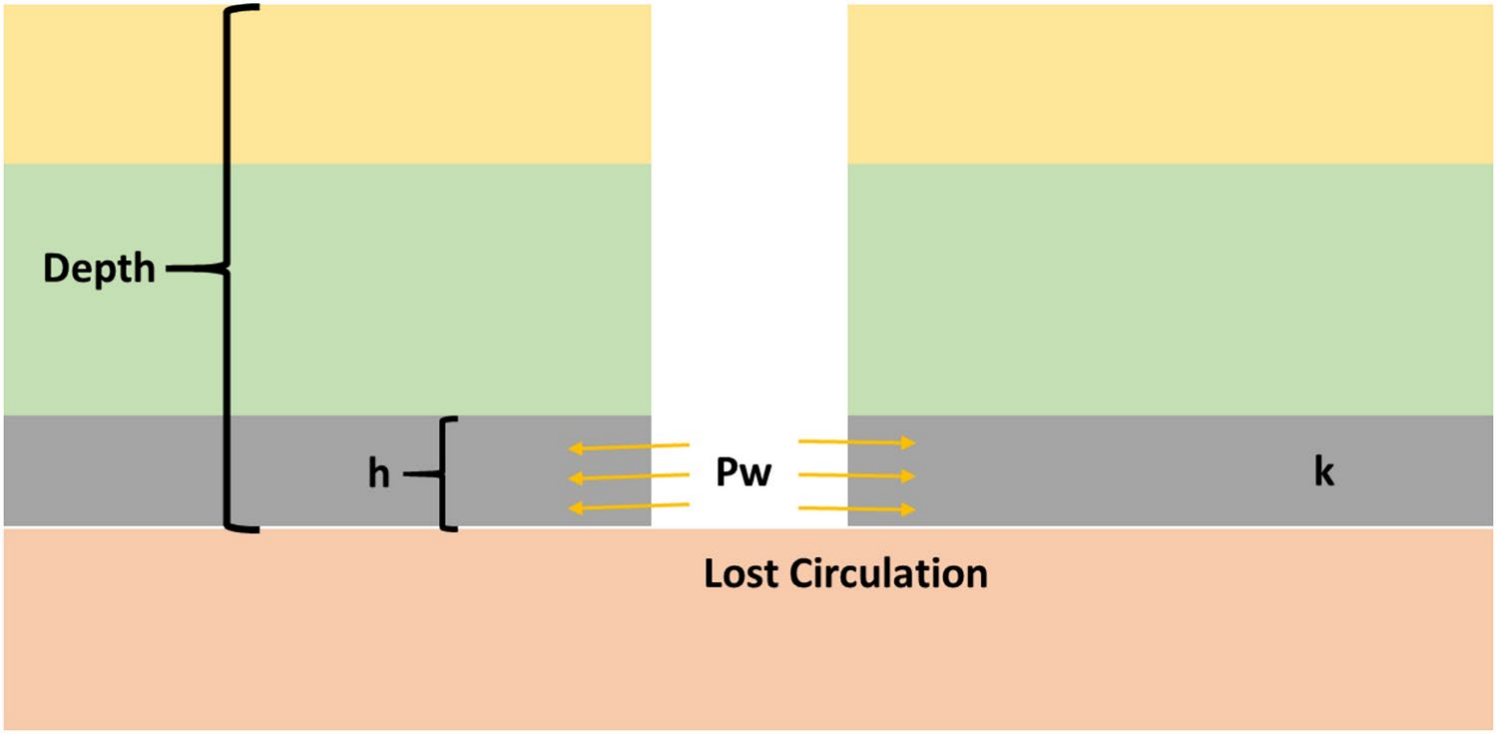
Lost circulation process (h: formation thickness, k: formation permeability, Pw: bottom-hole pressure).

As shown in Fig. 3, the drilling fluid loss process is similar to fluid injection in the porous medium. According to Darcy’s law, the loss rate depends on the parameters of bottom-hole pressure, formation pressure, viscosity of the drilling fluid, and the formation permeability. In this study reservoir simulator software (Eclipse E100) was used to simulate the drilling fluid loss process and generate mud loss data, which can be used to.

The data available in the drilling process include mud weight, mud viscosity, drilling depth, mud loss rate, formation type, and the thickness of the drilled formation up to that depth. Therefore, mud loss data were generated according to the following assumptions in the 810 data series:

1. Loss circulation limits: 1–250 bbl /hr.
2. Fluid type: water-based mud.
3. Increasing mud viscosity with increasing mud weight.
4. Increasing mud weight with increasing depth in general.
–10 layers with different pore pressure.

### Statistical analysis of generated data

**Fig. 4:**
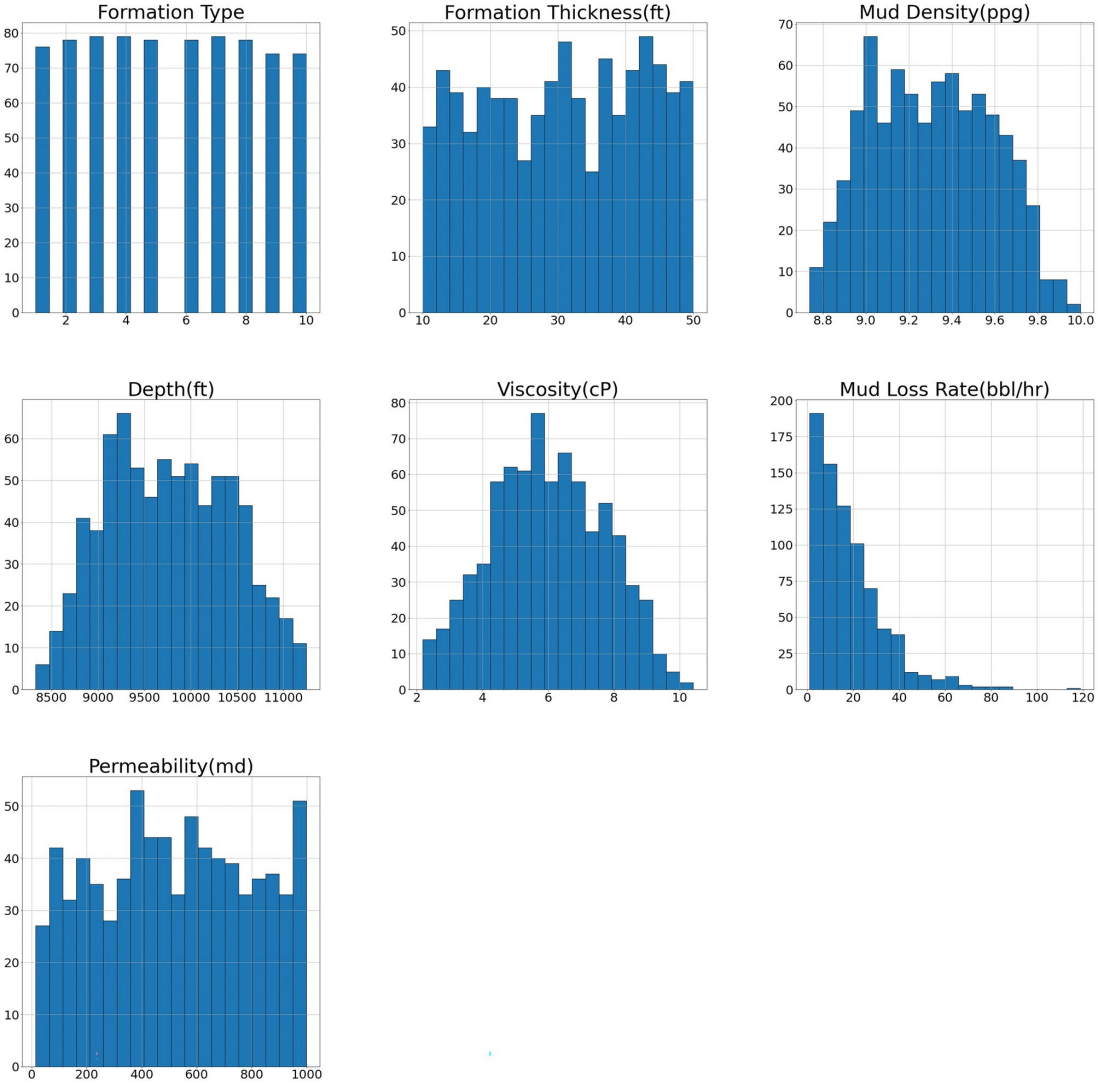
Histogram of mud loss data.

Data analysis of the mud loss dataset focused on definitive and inferential statistics which focused on univariate analysis. It was summarized the data by visualizing the distribution of each parameter in Table 2 and Fig. 4 shows the histogram of each variable.

**Table 2:** Data summary generated mud loss data.

	Formation type	Formation thickness (ft.)	Mud density (ppg)	Depth (ft.)	Viscosity (cP)	Mud loss rate (bbl/hr)	Permeability (md)
mean	5.467012	30.605016	9.305315	9764.129374	5.985918	18.102649	519.086477
std	2.850872	11.637433	0.279709	664.303636	1.712496	14.920821	278.588655
min	1	10.009243	8.737839	8327.332147	2.179200	1.008255	14.283200
25%	3	20.257640	9.076340	9223.384274	4.677512	6.949146	284.939012
50%	5	30.947308	9.305898	9746.420415	5.923977	14.018413	515.951805
75%	8	41.079716	9.524191	10,287.283320	7.282380	24.893571	749.618872
max	10	49.991703	9.997885	11,246.077410	10.410652	118.789042	998.041444
skewness	0.00862396	-0.05290973	0.089865	0.11015964	0.01782516	1.71079714	-0.0110721811
kurtosis	-1.21205184	-1.25240031	-0.917493	-0.86820517	-0.6640922	4.5967773	-1.1284377952

Mainly, the correlation coefficient (CC) is used to test the linear association between parameters. This can be expressed as follows:

1 $$CC = \frac{{\sum\limits_{i = 1}^{n} {(x_{m} - \overline{x}_{m} )(x_{p} - \overline{x}_{p} )} }}{{\sqrt {\sum\limits_{i = 1}^{n} {(x_{m} - \overline{x}_{m} )^{2} \sum\limits_{i = 1}^{n} {(x_{p} - \overline{x}_{p} )^{2} } } } }}$$

where n represents the number of experimental data, ${x}_{m}$, and ${x}_{p}$ define the measured and predicted parameters, respectively, and $\overline{x }$_m_, and $\overline{x }$_p_ signify their average values^58^.

**Fig. 5:**
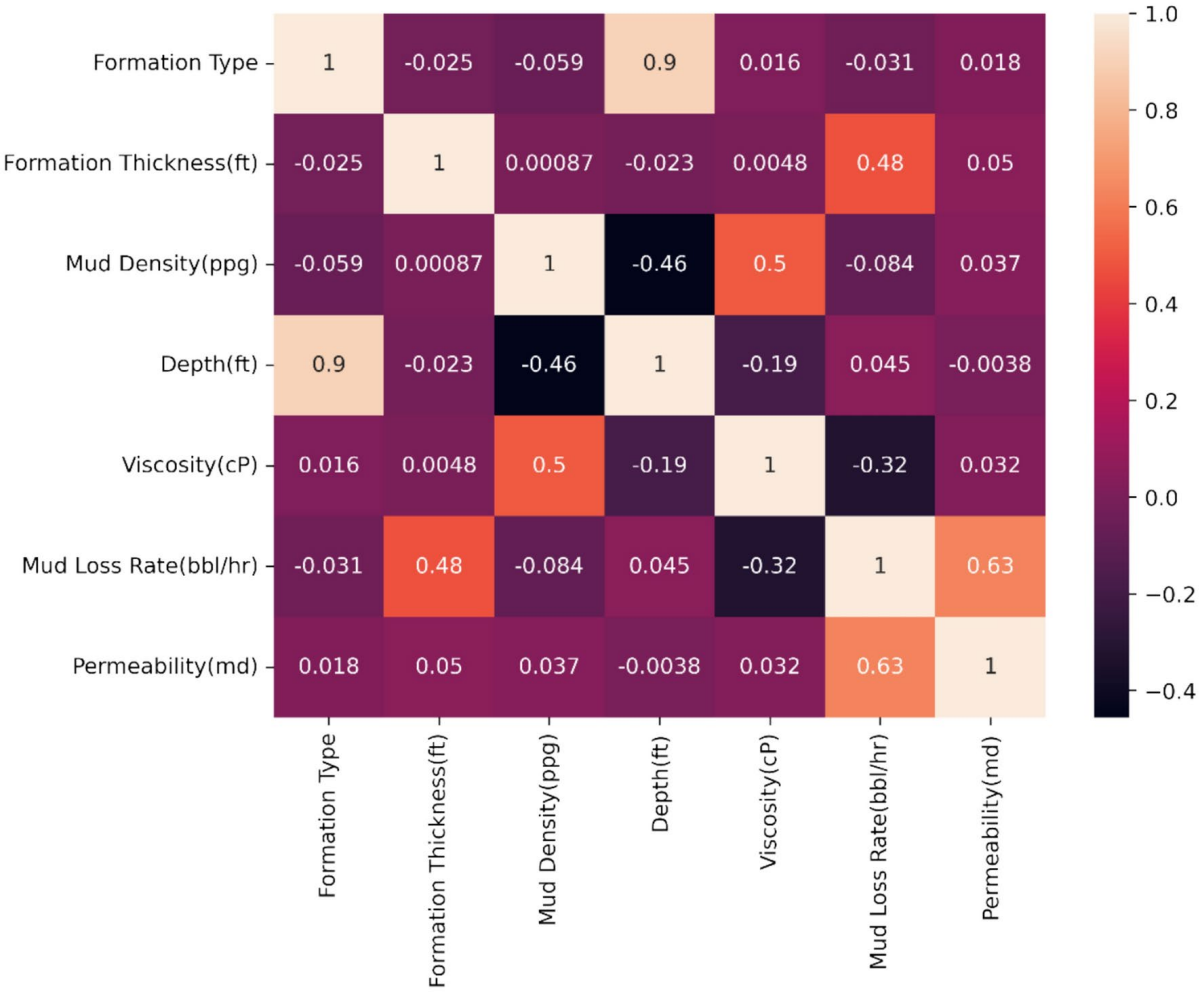
Correlation coefficient matrix for mud loss data.

Figure 5 indicates the CC matrix for analyzed variables. According to this data mud loss rate is the main parameter that influences the permeability parameter while other parameters have insignificant effects on the permeability. By analyzing the CC matrix, it can be seen that generated data is comparable to operational data, e.g. there is a high correlation between fluid viscosity and fluid density that is similar to the relation between these parameters in the real condition.

**Fig. 6:**
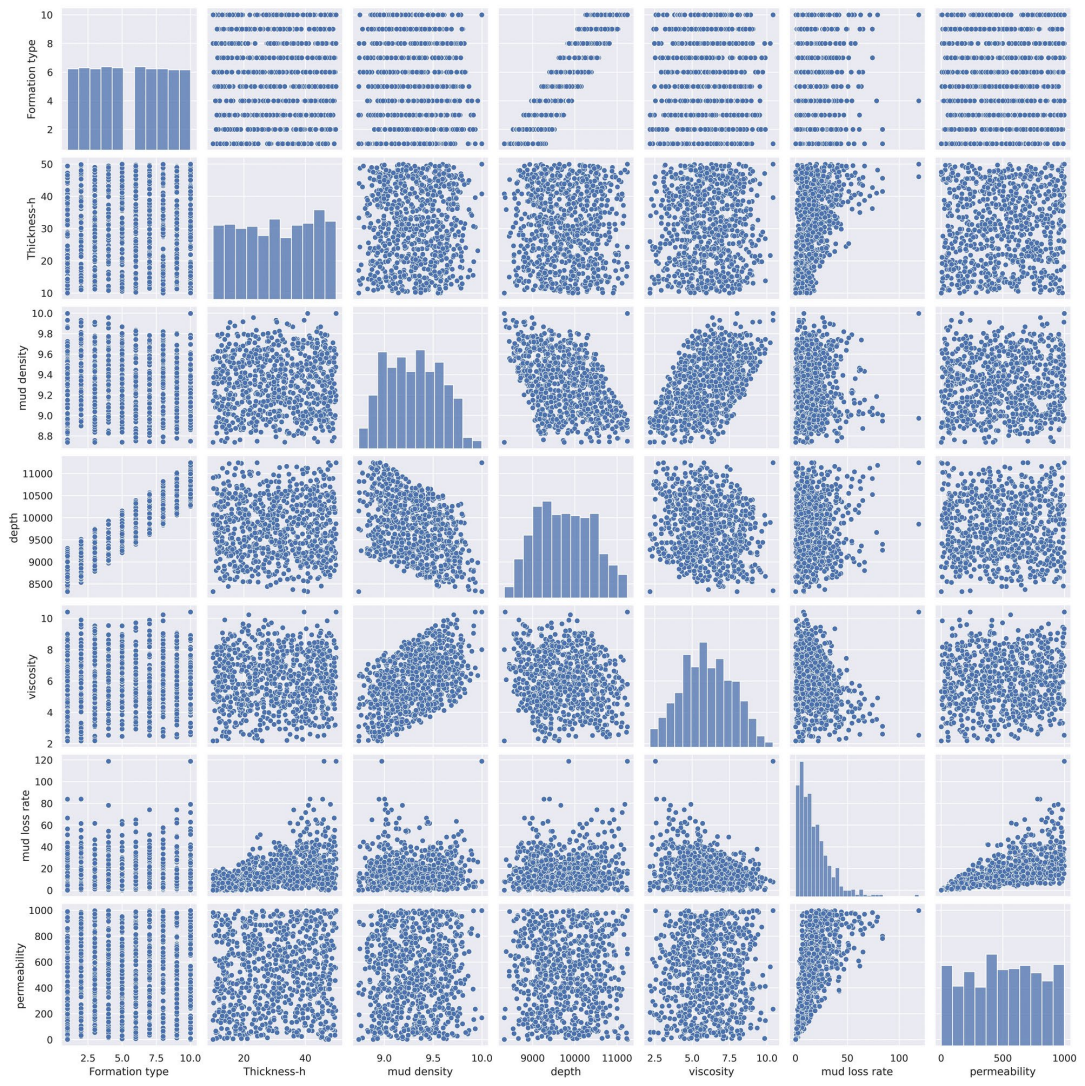
Non-linear relationships between variables.

Figure 6 shows non-linear relationships between variables. It is clear that the linearity relation between some variables, e.g. depth vs formation type, drilling fluid viscosity vs density, etc. Also, the non-linearity between some parameters is obvious, it is because of random conditions assumed for drilling conditions and no linear relationship between them at the real condition.

### Data arrangement

Mud loss data were passed through three steps before fitting into deep learning models. The process initiated with organizing categorical data, pre-processing the data using a normalization scaler, and splitting the data into training and testing sets.

The categorical variable organized in the data collection stage is formation types, which were nominated values 1 to 10, respectively. Then, normalization was performed to convert the variables between 0 and 1. Normalized data had an average of zero and a standard deviation of one. Such normalized data is used to train the model Neuropathy since it enhances learning processes and reduces high computational costs ^59^. The formula which used for normalization is as follows:

2 $$\overset{\lower0.5em\hbox{$\smash{\scriptscriptstyle\frown}$}}{x}_{i} = \frac{{x_{i} - x_{\min } }}{{x_{\max } - x_{\min } }}$$

In Eq. (2), x_i_ is the value of the variable for the i_th_ observation, x_min_ is the minimum value of a variable, and x_max_ is the maximum value of the variable. Finally, the reshaped data were split into two sets using 80:20 ratios for the training and testing set.

### Convolutional neural networks (CNN)

In the past decade, Convolutional Neural Networks have been responsible for breakthroughs in computer vision and image processing ^60–62^, obtaining state-of-the-art results on a range of benchmark and real-world tasks. More recently, one-dimensional CNNs have shown great promise in processing structured linguistic data in tasks such as machine translation ^63,64^ and document classification ^65,66^. Bai et al. ^67^ in 2018 indicated that, for many sequence modeling tasks, 1D-CNNs using current best practices such as dilated convolution often perform better than other recurrent neural network architectures.

**Fig. 7:**
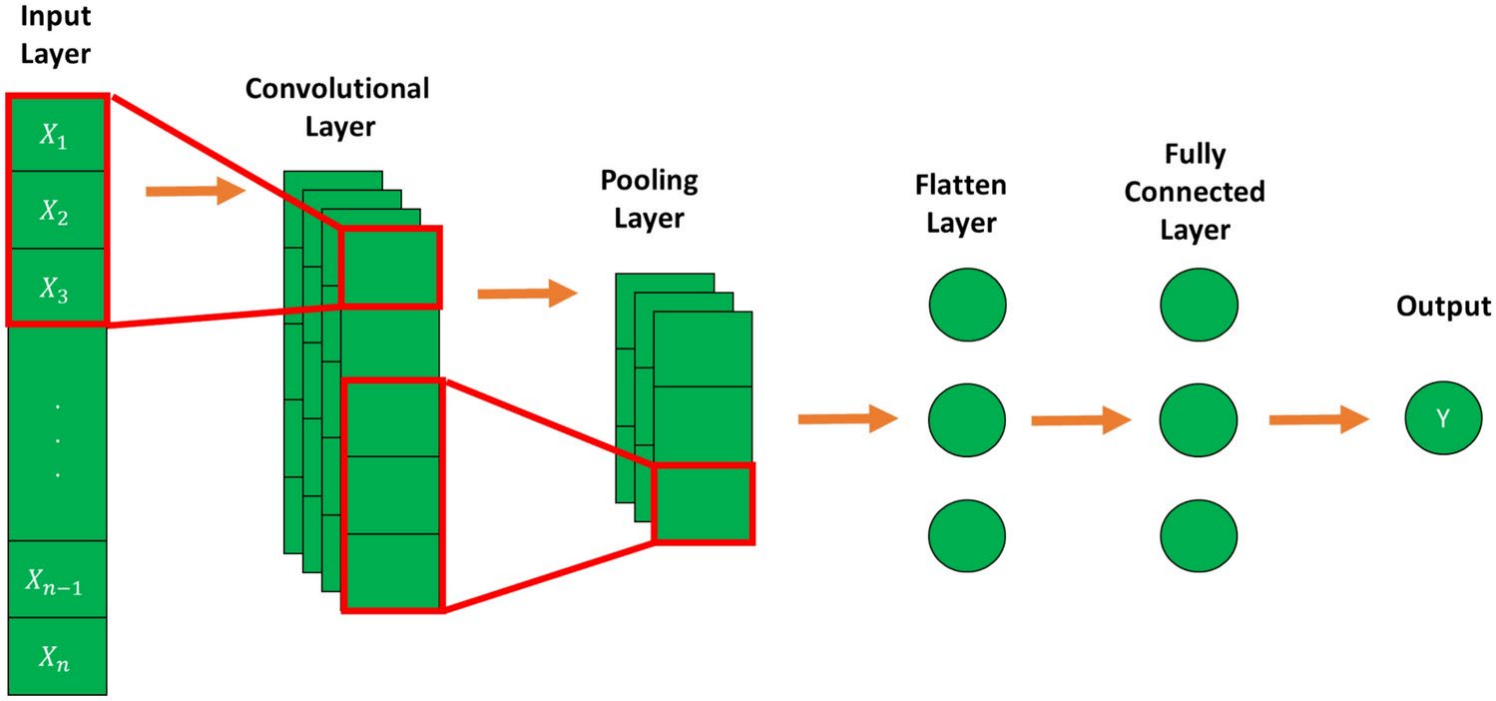
Example of One-dimensional convolutional neural network (1D-CNN) architecture.

A convolutional neural network is a type of feedforward neural network that consists of multiple convolution stages that perform the task of feature extraction and a single output stage that combines the extracted high-level features to predict the desired output ^68^. Figure 7 indicates a sample of the 1D-CNN architecture for the forecasting model.

In this study, Table 3 shows the elements of neural networks that included one 1D-CNN layer, one flattened layer, two dropout layers with a value of 0.2, and two fully connected layers. Exponential Linear Unit (elu) is applied in the convolution and fully connected layers as an activation function.

**Table 3:** Details of the used 1D-CNN model.

No	Layer (type)	Output shape	Parameter QTY
1	1D Convolution	(None, 5, 128)	384
2	Flatten	(None, 640)	0
3	Dropout	(None, 640)	0
4	Fully connected	(None, 64)	41,024
5	Dropout	(None, 64)	0
6	Fully connected	(None, 1)	65

### Deep jointly informed neural networks (DJINN)

The DJINN algorithm determines the appropriate deep neural network architecture and initializes the weights using the dependency structure of the decision tree trained on the data. The algorithm can be divided into three steps: building a set of decision trees, mapping the tree to a neural network, and fine-tuning the neural network through backpropagation ^69^.

#### Decision tree construction

The first step of the DJINN algorithm is to build a model based on a decision tree. This can be a single decision tree generating a neural network or an ensemble of trees, such as random forests^70^, which will create a set of neural networks. The depth of the tree is often limited to avoid creating neural networks that are too large; Maximum tree depth is a hyper-parameter that must be tuned for each data set^69^.

#### Mapping decision trees to deep neural networks

The DJINN algorithm selects a deep neural network architecture and a set of initial weights based on the structure of the decision tree. The mapping is not intended to reproduce a decision tree, but instead uses the decision path as a guide for network architecture and weight initialization. Neural networks are initialized layer by layer, whereas decision trees are typically saved for each decision pass. The path starts at the top branch of the tree and follows each decision to the left and then to the right until it reaches a leaf (prediction). Due to the way trees are stored, it is difficult to navigate the tree by depth, but it is easy to traverse the tree recursively. Mapping from a tree to a neural network is easiest if the structure of the tree is known before initializing the neural network weights. Therefore, the decision pass is executed twice; first, it determines the structure and then initializes the weights ^69^.

#### Optimizing the neural networks

As soon as the tree is mapped to the initialized neural network, the weights are adjusted using backpropagation. In this example, a deep neural network is trained with Google’s deep learning software Tensor Flow. The activation function used in each hidden layer is a modified linear unit, which generally works well in deep neural networks^71,72^ and can retain the values of neurons in previously hidden layers. The Adam optimizer^73^ is used to minimize the cost function (mean squared error (MSE) for regression, cross-entropy with logit for classification)^74^.

### Model performance evaluation

It is necessary to recognize the criteria associated with evaluating model performance. In this work, root mean squared error, mean absolute error, mean absolute percentage error, R-squared, and relative error were used as statistical indicators to evaluate the performance of the models.

#### Root mean squared error (RMSE)

The root mean squared error is used to see how well the network output matches the desired output. Better performance is guaranteed with smaller RMSE values. It is defined as follows ^75^:

3 $$RMSE = \sqrt {\frac{1}{n}\sum\limits_{i = 1}^{n} {(x_{m} - x_{p} )^{2} } }$$

#### Mean absolute error (MAE)

The mean absolute error is the average value of the absolute difference between the predicted value and the actual value. Errors showing a uniform distribution shall be presented. Furthermore, MAE is the most natural and accurate measure of the average level of error ^58^.

4 $$MAE = \frac{1}{n}\sum\limits_{i = 1}^{n} {\left| {x_{p} - x_{m} } \right|}$$

#### Mean absolute percentage error (MAPE)

The mean absolute percentage error is calculated by dividing the absolute error of each period by the observed values evident in that period. Then average these fixed percentages. This approach is useful when the size or dimensions of the predictor variable are important in assessing the accuracy of the prediction ^76,77^. MAPE indicates the degree of forecast error compared to the actual value.

5 $$MAPE = \frac{1}{n}\sum\limits_{i = 1}^{n} {\frac{{\left| {x_{p} - x_{m} } \right|}}{{x_{m} }}} \times 100\%$$

#### R-squared (R^2^)

An important index to check the correctness of the regression algorithm is ${R}^{2}$, which ranges from 0 to 1. ${R}^{2}$ is defined as follows^58^:

6 $$R^{2} = 1 - \frac{{\sum\limits_{i = 1}^{n} {(x_{p} - \overline{x}_{m} )^{2} } }}{{\sum\limits_{i = 1}^{n} {(x_{m} - \overline{x}_{m} )^{2} } }}$$

where n represents the number of observations, ${x}_{m}$, and ${x}_{p}$ define the measured and predicted parameters, respectively, and $\overline{x }$_m_ signifies the average of measured parameters.

### Relative error (RE)

The relative error is defined as the ratio of the difference of the predicted to the measured value. If ${x}_{m}$ is the measured value of a quantity, ${x}_{p}$ is the predicted value of the quantity, then the relative error can be measured using the below formula^78^.

7 $$RE = \frac{{x_{p} - x_{m} }}{{x_{m} }}$$

## Results and discussion

**Fig. 8:**
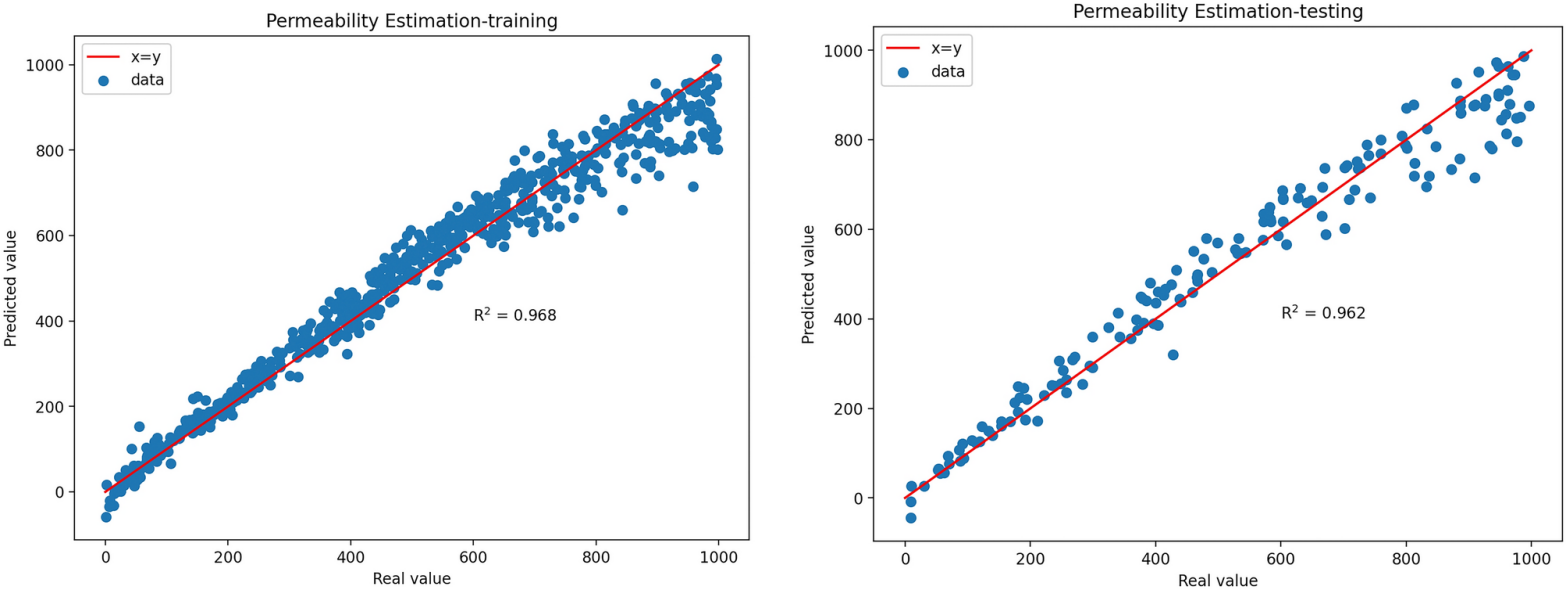
Cross-plots of the real value versus predicted values of the normalized permeability by 1D-CNN for training data.

**Fig. 9:**
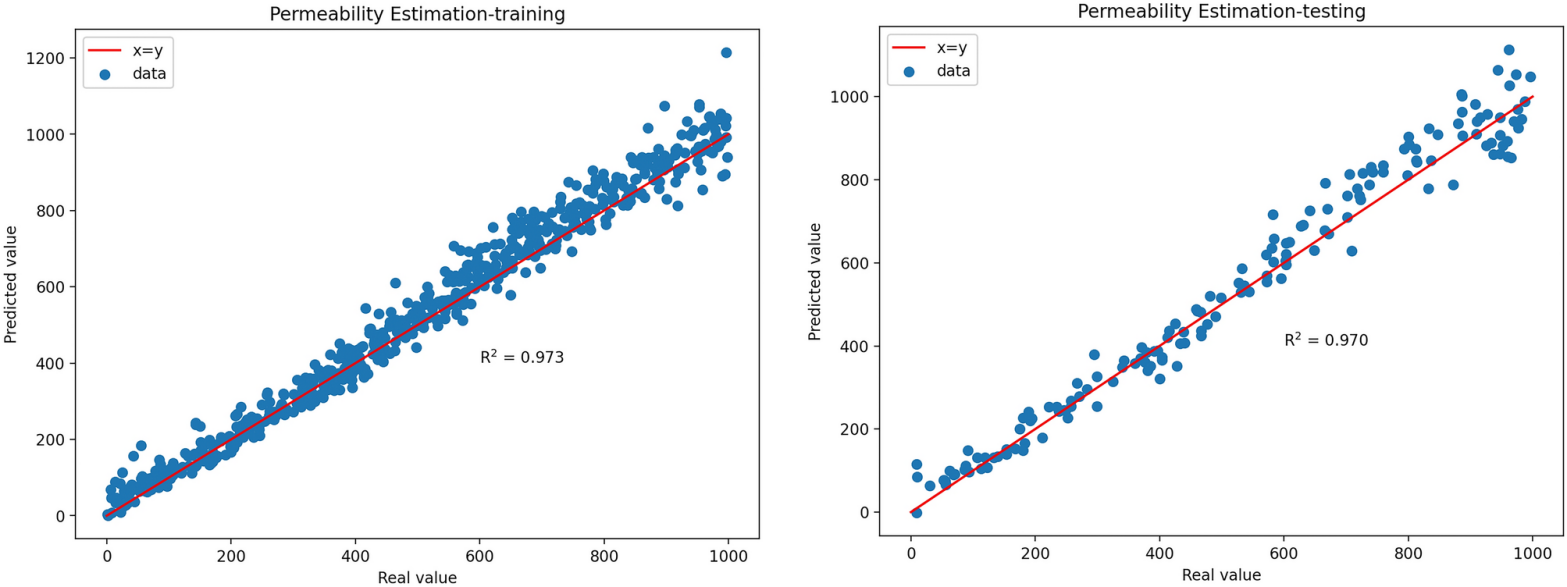
Cross-plots of the real value vs predicted values of the normalized permeability by DJINN for testing data.

Based on the previously mentioned methods fthe structural parameters of the CNN and DJINN for predicting formation permeability were determined, and the models were trained and tested. Real value versus predicted values of permeability (md) for training and testing data are displayed as cross plots in Fig. 8 and Fig. 9. R^2^ represents an alternative measure of forecast accuracy. As a precision indicator, it represents the proportion of the variance displayed by the dependent variable that can be predicted through the choice of the independent variable. If R^2^ = 1, this shows that the permeability of the formation can be predicted without error by the selected independent variables.

As can be seen from Table 4, the 1D-CNN prediction model has sufficiently high accuracy on training and test data (for training data: R^2^ = 0.968, RMSE = 50.78, MAE = 37.50, MAPE = 16.39; for test data: R^2^ = 0.962, RMSE = 58.17, MAE = 42.95, MAPE = 11.29). As shown in Table 5, the DJINN prediction model has also sufficiently high accuracy on training and test data (for training data: R^2^ = 0.973, RMSE = 46.15, MAE = 34.34, MAPE = 9.57; for test data: R^2^ = 0.970, RMSE = 51.39, MAE = 39.56, MAPE = 13.53).

**Table 4:** 1D-CNN model accuracies.

Accuracy	Training data	Testing data
R^2^	0.968	0.962
RMSE	50.78	58.17
MAE	37.50	42.95
MAPE	16.39	11.29

**Table 5:** DJINN model accuracies.

Accuracy	Training data	Testing data
R^2^	0.973	0.970
RMSE	46.15	51.39
MAE	34.34	39.56
MAPE	9.57	13.53

**Fig. 10:**
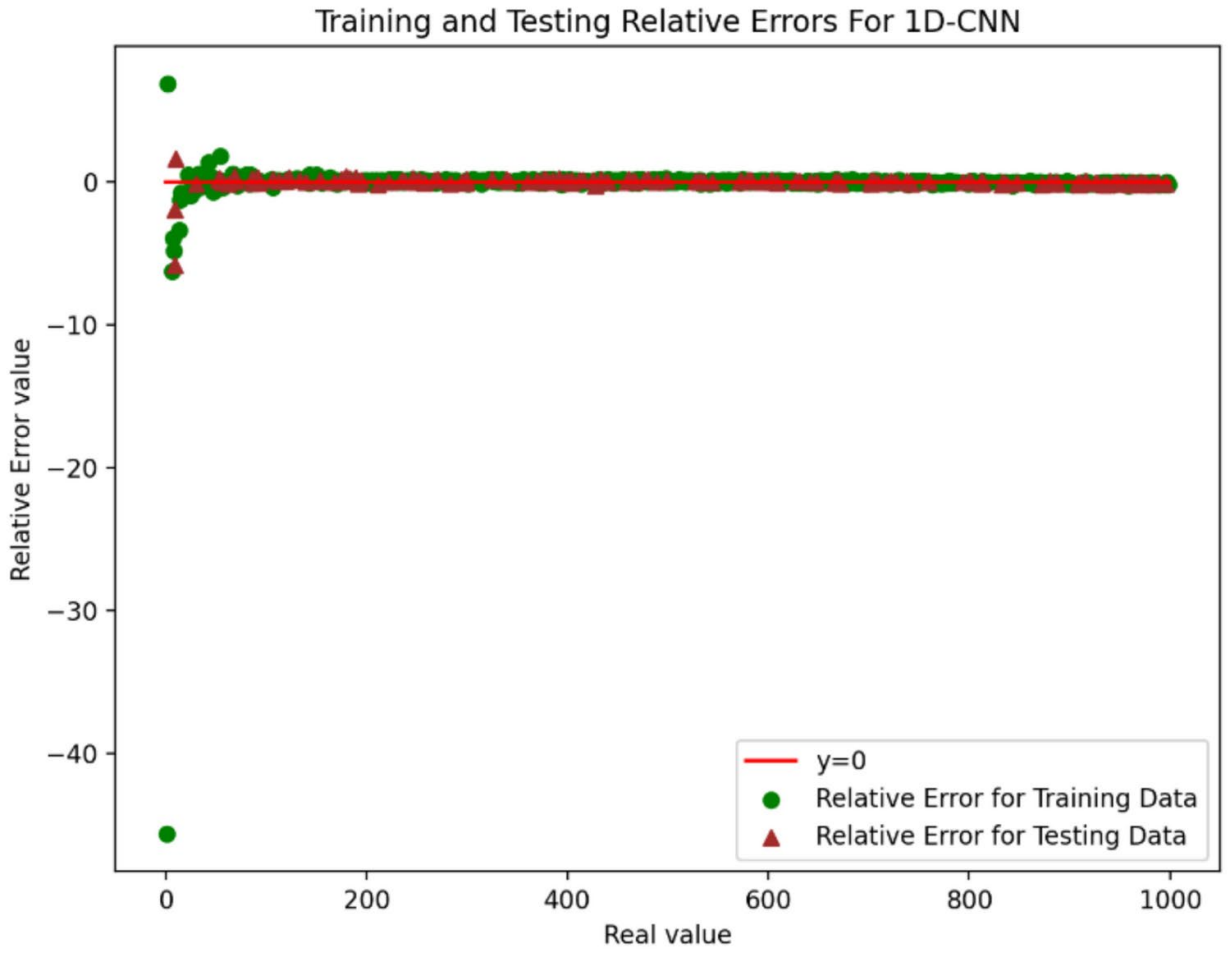
Training and testing relative error for 1D-CNN.

**Fig. 11:**
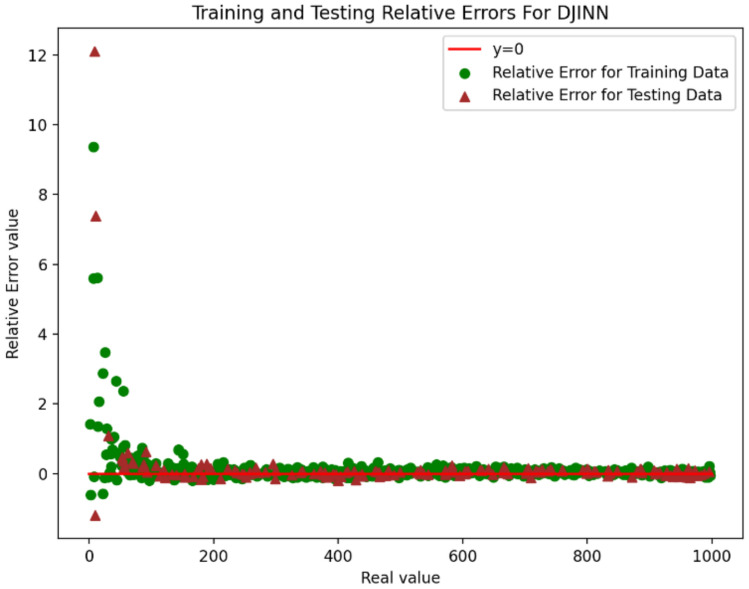
Training and testing relative error for DJINN.

Figure 10 and Fig. 11 indicate relative error for 1D-CNN and DJINN models. According to them the accuracy for data with low values is lower than the accuracy for data with high values. Therefore, these models are suitable for prediction data with high values.

**Fig. 12:**
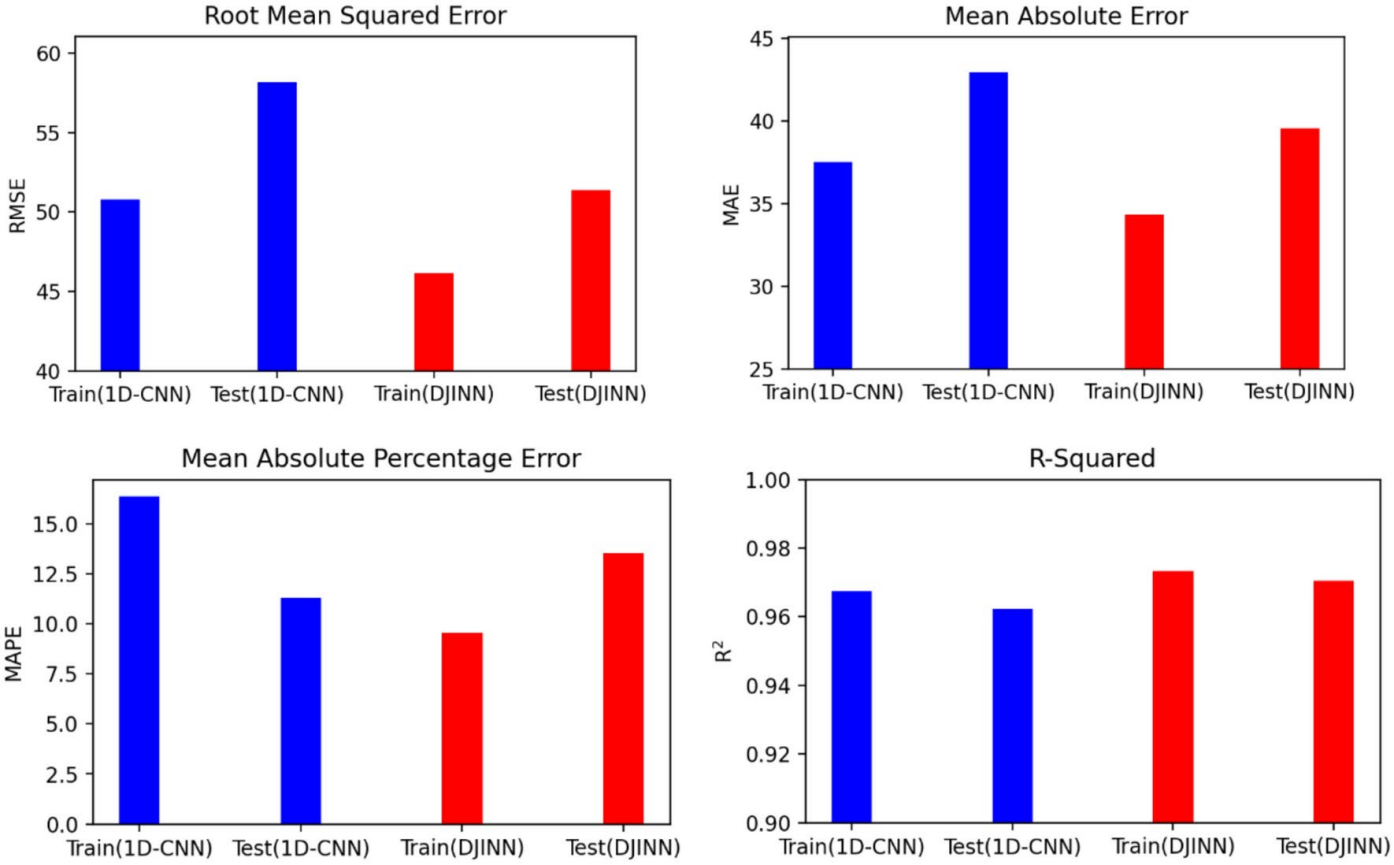
Comparing RMSE, MAE, MAPE, and R^2^ accuracies for training and testing data.

Figure 12 compares the computational error on training and test data for used algorithms. It indicates that RMSE, R^2^, MAE, and MAPE for the DJINN model are more accurate than the 1D-CNN model. Therefore, DJINN is a better algorithm for predicting formation permeability.

Table 6 compares the results of this study with those of recent permeability estimation studies. While most studies have used well log data, seismic data, rock imaging data, core data to estimate formation permeability by Analytical, statistical, and computational tools, and artificial intelligence tool, this study benefits from deep learning algorithms to estimate the formation permeability through mud loss data.

**Table 6:** Compare the results obtained with those of recent permeability estimation studies.

Item	Tools	Data type	References
1	Analytical, statistical, and computational tools	Well log data	^35,36^
2	Artificial intelligence	Well log data	^37,39,41–44^
3		Rock imaging data	^38^
4		Seismic data	^40,41^
5		Core data	^45^
6		Mud loss data	This study

## Conclusions

Permeability is the key parameter to reservoir characterization. There are various methods to evaluate the formation and estimate the formation permeability, but in some cases, the evaluation may not be done or it may not be done correctly.

This study estimated formation permeability using drilling fluid data and two deep-learning algorithms. Drilling data including depth, formation type, fluid density, fluid viscosity, formation thickness, and mud loss rate were generated by reservoir simulator software similar to real-world conditions and Deep learning algorithms including 1D-CNN and DJINN.

The results show that DJINN (R^2^ equals 0.973 on training data and 0.970 on test data) is a more accurate model than 1D-CNN (R^2^ equals 0.968 on training data and 0.962 on test data) in modeling this problem. Therefore, this study could present a novel method that uses mud loss data to estimate formation permeability accurately by deep learning algorithms (Supplementary table S1).

## Supplementary Information

Supplementary Information.

## Data Availability

"The authors confirm that the data supporting the findings of this study are available within the article and its supplementary materials."
