# Correction: Formation permeability estimation using mud loss data by deep learning

**DOI:** 10.1038/s41598-025-03476-9

**Published:** 2025-05-26

**Authors:** Yaser Abdollahfard, Seyed Morteza Mirabbasi, Mohammad Ahmadi, Abdolhossein Hemmati-Sarapardeh, Sefatallah Ashoorian

**Affiliations:** 1 Petroleum Engineering Department, Amirkabir University of Technology, Tehran, Iran; 2 Department of Petroleum Engineering, Shahid Bahonar University of Kerman, Kerman, Iran; 3 Institute of Petroleum Engineering, School of Chemical Engineering, University of Tehran, P.O. Box: 11155-4563, Tehran, Iran

Correction to: *Scientific Reports* 10.1038/s41598-025-94617-7, published online 30 April 2025

The original version of this Article contained an error in the Author contributions section. As a result,

“Yaser Abdollahfard authored the main manuscript text, produced Python code for result output and modeling, and created artificial data using reservoir simulation software. Dr. Seyyed Morteza Mirabbasi revised the manuscript technically as drilling engineer. Dr. Mohammad Ahmadi revised the manuscript as Author corresponding. Dr. Abdolhosein hemmati revised the manuscript as data scientist. Dr. Sefatollah Ashorian revised the manuscript grammarly.”

now reads:

“Yaser Abdollahfard authored the main manuscript text, produced Python code for result output and modeling, and created artificial data using reservoir simulation software. Dr. Seyyed Morteza Mirabbasi revised the manuscript technically as drilling engineer. Dr. Mohammad Ahmadi revised the manuscript as corresponding author. Dr. Abdolhosein Hemmati revised the manuscript as data scientist. Dr. Sefatollah Ashorian revised the manuscript as a final technical check reviser.”

The original Article has been corrected.

